# Testicular Cancer: Genes, Environment, Hormones

**DOI:** 10.3389/fendo.2019.00408

**Published:** 2019-07-02

**Authors:** Luca De Toni, Iva Šabovic, Ilaria Cosci, Marco Ghezzi, Carlo Foresta, Andrea Garolla

**Affiliations:** ^1^Unit of Andrology and Reproductive Medicine, Department of Medicine, University of Padova, Padova, Italy; ^2^Department of Clinical and Experimental Oncology, IOV-IRCCS, Padova, Italy

**Keywords:** suscepibility genes, temperature, endocrine discruptors, disorders of sex development, GWAS

## Abstract

Testicular cancer (TC) represents one of the most peculiar clinical challenges at present. In fact, currently treatments are so effective ensuring a 5 years disease-free survival rate in nearly 95% of patients. On the other hand however, TC represents the most frequent newly diagnosed form of cancer in men between the ages of 14 and 44 years, with an incidence ranging from <1 to 9.9 affected individuals per 100,000 males across countries, while the overall incidence is also increasing worldwide. Furthermore, cancer survivors show a 2% risk of developing cancer in the contralateral testis within 15 years of initial diagnosis. This complex and multifaceted scenario requires a great deal of effort to understand the clinical base of available evidence. It is now clear that genetic, environmental and hormonal risk factors concur and mutually influence both the development of the disease and its prognosis, in terms of response to treatment and the risk of recurrence. In this paper, the most recent issues describing the relative contribution of the aforementioned risk factors in TC development are discussed. In addition, particular attention is paid to the exposure to environmental chemical substances and thermal stress, whose role in cancer development and progression has recently been investigated at the molecular level.

## Introduction

With an overall annual incidence of nearly 1% among all newly diagnosed cancers in males, testicular cancer (TC) represents the most common tumor in men at the age ranging between 14 and 44 years, which is considered to be the fully working/reproductive age (1, 2). Before the 1970s, the mortality rate for TC was extremely high due to the metastatic degeneration of the disease, whilst the only two treatments to contain the risk of relapse were the retroperitoneal lymph node dissection, associated or not with radiotherapy. Thereafter, the development of an effective chemotherapy changed the “rules of the game.” In fact, the current multidisciplinary approach to the treatment of TC, comprising surgery and adjuvant chemo- or radiotherapy, results in a 5 years survival rate of >95%. As a consequence, TC is now considered as a model for a curable cancer (3).

In spite of these indisputable progresses, TC still presents multi-leveled challenges that should not allow our guard to be lowered:

- Epidemiological evidence: the annual incidence of TC has doubled over the past 40 years with an increasing trend over time, particularly in Caucasian males (4). Indeed, data from the African and Asian continents show an incidence lower than one case every 100,000 males, whilst Scandinavian countries report the highest rate of newly affected individuals worldwide (from 9.4 to 9.9 males every 100.000 males). This trend by ethnicity is further confirmed by data from United States where TC is found more frequently in white males compared to African Americans (1.2 vs. 6.9 affected individuals per 100.000 males, respectively) (5, 6).
- Clinical evidence: previous history of TC, even if properly treated and monitored, represents a major risk factor for a second contralateral cancer. The overall risk for a secondary TC is approximately 5% within 5 years from diagnosis, and in most cases presenting within 2 years from the first diagnosis (7). In this regard, primary testicular size and the degree of invasion of the rete testis are considered two major prognostic factors for relapse (8, 9).
- Therapeutic evidence: in spite of the aforementioned effectiveness, the treatment of TC itself is frequently associated with an increased risk of developing long lasting adverse effects like infertility, hypogonadism, metabolic/cardiovascular derangements, and osteoporosis, which actually represent the most relevant life threatening consequences of the TC therapy (10– 12).

For all these reasons, the identification of pathogenic mechanisms and risk factors involved in testicular carcinogenesis still represent topics of extremely high clinical interest. Indeed, the probability of developing TC is the result of a combination of a number of factors that can be distinguished, in general terms, into genetic, environmental, and hormonal factors.

## Genes

The fact that TC development relies on genetic factors is widely acknowledged. Despite the fact that 90% of males affected by TC have no previous familiar cases of this disease, population-based studies in the late 1990's-early 2000's showed that having a brother with an history of TC increases the risk of the disease from 8- to 10-fold, compared to the general male population. On the other hand, having a father affected by TC increases the relative risk for the male child from four- to 6-fold (13–15). In 2002, a pioneering study on a population-based registry, evaluating 9.6 million individuals from the nationwide Swedish Family-Cancer Database, attempted to distinguish between the respective genetic, pure-environmental and childhood-environmental contribution to the development of cancers, essentially based on epidemiologic considerations (16). Interestingly, TC resulted as one of the most associated neoplasms with genetic factors (25%), right after the thyroid (53%), and endocrine glands in general (28%). In addition, a recent study on a population-based registry, evaluating monozygotic and same-sex dizygotic twin individuals disclosed an esteemed familiar risk of heritability for TC of nearly 40% with a significant portion, however, attributable to shared environmental conditions (17).

In spite of the clear evidence supporting the genetic background in TC development, the availability of reliable studies providing qualitative and quantitative data about the genetic basis of familial TC still represents a major challenge. In 2006, a linkage study on 237 pedigreed families, with a history of one or more cases of TC, identified six regions of interest on chromosomes 2p23, 3p12, 3q26, 12p13-q21, 18q21-q23, and Xq27 as susceptibility loci. However, further widenings showed that no single locus accounted for the majority of the familial aggregation observed in TC, suggesting at the same time a major role of multiple susceptibility loci with singular weaker effects (18). To this regard, significant advances have been provided by the availability genome wide association studies (GWAS) that, since the mid-2000's, progressively increased the number of susceptibility loci with a predicted effect on TC development (19–24). In a recent GWAS and meta-analysis, comprising more than 5,500 cases and 19,000 controls from northern Europe, (25) identified and confirmed 44 independent TC risk loci (19 newly discovered and 25 previously reported). Interestingly, through a complex *in situ* chromosome conformation-capture analysis in TC cells, a tentative model of chromatin interactions between predisposition SNPs and target genes was performed, identifying three possible pathogenic mechanisms. In particular, 10 of the risk loci contained genes associated with the transcriptional regulation of cell development such as *GATA4* and *GATA1* genes. These are transcription factors involved in the specification and differentiation of postnatal testicular development, whose risk alleles polymorphisms have been previously associated with tumor progression (26–31). A significant association was also found for *PRDM14* and *DMRT1* genes, involved in germ cell specification-sex determination, and the *SALL4* gene through the disruption of the *POU5F1* binding motif (32–35), the latter associated with the maintenance of pluripotency in embryonic stem cells (36). In addition, five TC risk loci were associated with candidate genes with roles in microtubule and chromosomal assembly, particularly the *TEX14* gene, involved in kinetochore-microtubule assembly in the testicular germ cells (37–39), the *WDR73* gene, encoding a key protein for microtubule organization during interphase (40), and the microtubule assembly–related genes *PMF1, CENPE*, and *PCNT* (41–44). Furthermore, three TC risk loci subtended a major role of KIT–MAPK signaling, in agreement with recent evidence showing the *KIT* gene as a major somatic driver for TC development (24). In clinical terms, the 44 identified risk loci for TC accounted for 34% of the father-to-son familiar risk for TC development, whilst the top 1% genetic risk at a polygenic risk scores model had a relative risk of 14% and a 7% lifetime risk of developing TC (25). However, this pattern is likely to be widened, thanks to the increasing number of GWAS successively issued. A very recent meta-analysis of five available GWAS, including the X-chromosome, identified further 12 risk loci associated with TC, highlighting the possible involvement of additional cell pathways in TC, such as germ cell development and pluripotency through the *TFCP2L1* and *ZFP42* genes, the kinetochore function through the *ZWILCH* gene, the response to DNA damage through the *TIPIN* gene and the mitochondrial function through the *TKTL1* and *LHPP* genes (45).

From this brief summary, it is clear that the pathogenesis of TC relies on a wide spectrum of genetic factors. Recently, a great deal of interest has been sparked by the role of gene copy number variations (CNVs) in cancer development and, particularly, in TC (46–48). In this regard, our group recently investigated the involvement of the *E2F1* gene CNVs as a TC risk factor (49). As a member of the E2F protein family, E2F1 is a transcription factor that regulates the transition of the cell cycle from the G1 phase to the S phase, through an interaction with the retinoblastoma tumor suppressor (RB) protein (50–52). Deregulation of E2F1-pRB binding increases the access of E2F1 to E2F1-binding target genes, containing the E2F-binding site, and this is thought to increase the susceptibility of tumor development (53). Importantly, experimental overexpression of E2F1 in carcinoma cell lines has been associated with increased cell proliferation through the mTOR signaling pathway (54). Interestingly, in our study group of the 261 patients with an history of testicular germ cell tumors and the 165 controls, we found duplications of the *E2F1* gene only in TC patients with a global prevalence of 6.5 percent. This was associated with the increased expression of the E2F1 protein only in tumor tissue specimens obtained from those patients harboring three copies of E2F1, whilst surrounding non tumor-tissue showed both lower E2F1 protein expression and downstream-mTOR phosphorylation (49). These results are highly suggestive of an involvement of E2F1 CNVs in TGCT susceptibility through the Akt/mTOR signaling pathway.

It should also be noted that several risk factors clinically associated with TC development, largely rely on genetic factors. Cryptorchidism, the failed descent of the testis in the scrotum through the inguinal canal during the embryonic life, affects 2–9% of boys born full term and is associated with an almost 9-fold increased risk of TC, compared to the general population (55, 56). The migration process of the embryonal testis can be functionally divided into two sequential phases: the trans-abdominal phase and the inguino-scrotal phase (57). Data from animal models disclosed that each phase is finely regulated by specific factors. In particular, the transabdominal migration of the testis depends primarily on insulin-like peptide 3 (INSL3) and its receptor, RXFP2 (58–60), whilst the inguino-scrotal phase largely depends on androgens signaling (61, 62). Genetic screening in cryptorchid boys showed, respectively, a 2 and 4% prevalence of mutations in the *INSL3* and *RXFP2* genes, more frequently in bilateral forms, whilst there is less agreement for a causative role of polymorphic variants (63–65). Interestingly, there is poor association between mutations of the *AR* gene and isolated cryptorchidism since the prevalence in cryptorchid males is generally lower than 2% (63, 66). In addition, expansion sites in the first exon of the *AR* gene, also known as poly CAG and GGN repeats, are acknowledged as a modulator of AR transactivation activity but their causative role in undescended testis is still under debate (67, 68).

With regards to cryptorchidism, one of the most relevant causes of this clinical condition is a chromosomal alteration such as Klinefelter syndrome (KS), affecting ~1 in every 700 men (69). KS patients typically present with small testes, infertility, high levels of gonadotrophins and testosterone (T) at the lower levels of normality, whilst cryptorchidism presents nearly six times more frequently than in the general male population (69). The existing literature relating KS and TC, principally Leydig cell tumor, is quite abundant and mainly rely on case reports, however no conclusive association has been provided by the few available epidemiological studies on larger cohorts (63, 70–84). Hence, further studies are required to clearly identify the relative risk of TC associated to KS.

Other genetic causes of isolated cryptorchidism are ascribed to mutations of the AMH gene or its receptor in the persistent müllerian duct syndrome described below (85, 86). In addition, hypospadias, the urethral malformation during embryonal penis development, is also considered a risk factor for TC (87). In particular, hypospadias accounts for about 10% of familial clustering, whilst the estimated heritability of this disease ranges from 57 to 77% (88, 89).

## Environment

The identification of direct environmental causes of TC development represents a problem with higher complexity. In fact, most of the acknowledged tumorigenic physical or chemical agents act indirectly through the disruption of the hormonal circuits regulating testis function, or by influencing the function of susceptibility genes (90). However, according to available literature, exclusive environmental risk factors for TC can be formally distinguished into four main classes: microbiological, mechanical, chemical, and physical.

### Microbiological

Epidemiological data in 2002 estimated viral infections to be the causative role of ~12% of cancers worldwide (91). In particular, the pathogenic role of infectious agents in testis tumors has been hypothesized since the late 1980s. Based on epidemiological similarities between Hodgkin's disease and TC, Algood et al. (92) investigated the possible causative role of early exposure to the Epstein-Barr virus (EBV), through the evaluation of antibodies to the EBV capsid antigen, in a small group of patients with an history of stage I germ cell tumors of the testes, receiving surveillance after orchiectomy (92). Interestingly, 80% of patients showed elevated titers for anti EBV antibodies compared to the control subjects, strongly linking cancer disease to previous viral exposure. In 1994 (93) further investigated the detection of EBV-DNA in testis specimens from patients with testicular germ cell tumors, including preinvasive carcinoma *in-situ*. A weak positivity for EBV DNA was detected in only six out of the 20 samples but none of the specimens showed a positive staining at either anti EBV-immunohistochemistry or *in situ*-hybridization techniques, ruling out a direct involvement of EBV and rather suggesting a putative growth-stimulating role of EBV-transformed lymphocytes infiltrating in testis stromal tissue (93). In 2013 Yousif et al. (94) aimed to quantify the possible association between viral infections and TC through a meta-analysis. Interestingly, serological markers of exposure to EBV, Cytomegalovirus, and Parvovirus B19 were associated with TC with pooled odd ratios (OR) of respectively, 4.80, 1.85, and 2.86. Particularly for Human-immunodeficiency virus (HIV), authors first identified a pooled OR of 1.79 (94). This evidence was subsequently confirmed by several studies showing a relative risk ranging from 0.7 to 3.1 [reviewed in Hentrich and Pfister (95)]. However, as for EBV, a clear mechanistic model explaining the association between TC and HIV is currently under debate.

### Mechanical

Despite being poorly acknowledged among typical risk factors for TC, mechanical, and particularly traumatic events on the testis, are considered a causal factor of this disease. Indeed, the experimental model of intra-testicular hematoma induced by injection of an autologous blood equivalent in rat testis was associated with a significant and long-lasting alteration of the testis structure, such as the reduction of the overall testis volume and the reduction of the seminiferous epithelium size. All these features resulted in altered testis function such as the under-representation of the germ cell population within the seminiferous tubule, altered sperm parameters, and a trend toward lower testosterone levels (96). Based on this profound alteration of the testis' functional architecture, the degeneration into cancer is rather intuitive, particularly in those situations of prolonged though subclinical testis trauma. In spite of this simple model, available evidence linking testis trauma to cancer are sparse and/or non-conclusive.

An interesting study from Dusek et al. (97) aimed to quantify the contribution and mutual interactions of very different types of potential risk factors for TC through the administration of standardized questionnaires to patients recruited in two Czech cancer centers, compared to healthy and age-matched controls (97). Interestingly, in addition to acknowledged risk factors like cryptorchidism and testis atrophy, a significant association was found for testicular trauma resulting with a nearly doubled risk for TC compared to the controls.

Another example of this model is represented by prolonged testis-micro traumas in patients practicing sports. In fact, testicular derangements such as testicular torsion, epididymitis, and testicular tumors are frequently observed by medical sport physicians (98). Coldman et al. (99) showed that cycling, particularly during teenage years, was associated with an almost doubled risk for TC even after correction for confounding factors like cryptorchidism or inguinal hernia. Also, horse-riding resulted in a nearly 3-fold greater risk for TC, which remained substantially unaltered after correction for confounding factors, whilst no significant association was reported for motorcycling or soccer (99). However, subsequent studies failed to identify a significant association between these sports with TC, suggesting further investigation (100, 101).

### Chemical

Available data on chemicals exerting a direct role as a risk factor for TC mainly derive from occupational studies. Particular attention has generally been drawn by the exposure to heavy metals in extracting and processing plants. Heavy metals, most frequently absorbed as organometallic compounds, are known to accumulate in tissues, both disrupting their biological functions and representing long-term reservoirs, resulting in prolonged exposure to metal pollutants (102). In particular, transition series-metals like cadmium (Cd), mercury, and cobalt are acknowledged as carcinogens from several experimental studies performed in both animal and cell models (103–105). However, a direct association between Cd exposure and TC is still under ascertainment. In 2011, an epidemiological study for cancer incidence was performed in the Kempen area across the Dutch-Belgian border, featuring the very long activity of cadmium and zinc smelters. Compared to the control population, identified through regional population-based cancer registries, environmental exposure to Cd showed an increased risk for female lung cancer, male and female bladder cancer and prostate cancer but not TC (106). Similarly, another study focused on the north-east Belgium area investigated the ~17 years incidence of cancers, finding an overall increased risk of doubling the 24-h urinary cadmium excretion, however no significant association with TC was documented (107). On the other hand, previous studies performed on metal workers in the Hannover region of Germany showed a nearly doubled risk of developing TC compared to the aged matched healthy controls, but no single chemical emerged at significant levels from the association analysis (108). In addition, Norwegian metal workers working with ferrosilicon and silicon furnaces showed a more than doubled incidence of TC compared with the estimated incidence in the general Norwegian population according to the age and historical period (109).

Another class of environmental chemicals associated with TC is pesticides, as depicted by epidemiological studies disclosing an increased incidence of TC in agricultural employees (110–112). However, two great meta-analysis in 1992 and 1998, respectively, failed to recognize a significant risk due to the exposure to pesticides in farmers (113, 114). To this regard, opportune distinctions should be made since substantial difference exists among countries in terms of chemicals, formulations, and regulatory principles (90). Furthermore, great differences in terms of toxicological effects of the different molecule classes is likely to exist in humans. In fact, organochlorines pesticides are supposed to act as endocrine disruptors (see below) whilst pyrethroids are likely to exert a direct effect on the cell cycle (115, 116).

### Physical

Among those physical risk factors theoretically associated with an increased risk of TC, such as the exposure to ionizing radiations, ultraviolet light and electrical work, the most clinically valued is exposure heat stress. As depicted by the external location of male genitalia, the proper germ cell maturation within the seminiferous tubule is maintained at 2–8°C below the body core temperature (117). Systematic exposure of the testis to over-physiological temperatures has been associated with several, and generally reversible, testis derangements such as a reduced sperm count, motility, mitochondrial function, and even altered sperm membrane composition (118, 119). As for other environmental factors, the possible association between heat exposure and TC was investigated through occupational studies. Early studies in 1995 performed on TC patients and healthy age-matched controls, revealed that occupational exposure to high or extreme temperatures was associated with an adjusted OR of respectively, 1,2 and 1,7, suggesting external temperature as an independent risk factor (120). A subsequent study in 2001 confirmed that the standardized incidence ratio for TC in fire fighters was 3,0 with no increased risk from any other cause of death (121). However, exposure to milder heat stress like showering and bathing was not associated with any significant risk for TC (122).

## Hormones

Exactly like other endocrine tissues in the body, the testis is both the target and the source of hormones strictly linked in a feedback-loop regulating pathway (123). In particular, the activity of the hypothalamus/pituitary/gonadal axis takes place from the early phases of embryo development, regulating testis descent and, the adequate location within the scrotal sac and the proper spermatogenic and endocrine functions, whose systemic effects are well-known (124). The early disruption of this hormonal circuit reverberates on testis function in adult life and represents a major risk factor for TC. Indeed, a tentative mechanistic model of the neoplastic transformation of germ cells has been developed by Rajpert-De Meyts et al. (125). This hypothesis is based on the strict similitude between primordial germ cells and gonocytes with tumor cells of the carcinoma *in situ* (CIS), verified at the molecular level by the shared expression of genes involved in pluripotency and proliferation, such as *NANOG, STELLAR, DPPA-5, GDF3, K-RAS*, and *CCND2* (126–129). In this context, the delayed development of germ cells, associated with long-term maintenance of embryonic genes, would represent a key event for the subsequent degeneration into cancer cells (130, 131). The disruption of the hormonal milieu of germ cells would then result in misleading signals altering the cell phase-switch toward mitosis and meiosis, with the consequent risk of a neoplastic transformation in adult life (125).

The most studied model of hormonal risk factor for TC is the disorders of sex development (DSD) in 46, XY males, frequently associated with androgen-insensibility syndrome (AIS), further distinguished into complete (CAIS), partial (PAIS), or mild (MAIS) forms (132). As can be guessed from the name of this pathological condition, its clinical characteristics range from a female phenotype of CAIS, in spite of an XY karyotype and normal androgen production, to severe under masculinization in PAIS, such as female external genitalia or hypospadias or micropenis, or male infertility and/or gynecomastia in MAIS. A general feature of the different forms of AIS is the altered function of the androgen receptor, resulting in a resistance to androgens as activating ligands. Genetic variants of the *AR* gene are commonly acknowledged as being causative of AIS. In particular, 95% of CAIS are associated with inactivating mutations of AR. In PAIS however, mutations of the *AR* gene are detected in <25% of patients whilst a complementary causative role has been ascribed to genetic variants of the protein and cofactors that concur to the AR signaling pathway, such as the deficiency 17β-hydroxysteroid dehydrogenase (17β-HSD), a key enzyme in steroidogenesis (133, 134). Also, persistent müllerian duct syndrome (PMDS) is a form of disorder of sex differentiation in 46, XY males caused by an inactivating mutation of the gene for AMH/MIS (45% of cases) or its type II receptor (39% of cases) (135). PMDS patients present genotypically and phenotypically as males with unilateral or bilateral cryptorchidism and/or an inguinal hernia at infancy (136). In general, DSD, and in turn AIS, are associated with and increased risk of TC with an estimated overall prevalence of nearly 5.5 percent, ranging from 0.8% in CAIS-associated DSD, 15% in PAIS to 17% in 17β-HSD deficiency (137, 138). Despite the fact that TC in PMDS has been described in several case reports, association studies on large cohorts are actually not available (139–145). Of note, testis retention in the abdomen represents an association with DSD and represents by itself a risk factor of TC as previously discussed. Interestingly, the association between TC and cryptorchidism has been documented in DSD from both AR mutation and PMDS. Thus, a major pathogenic role of DSD-associated testis retention in TC cannot be ruled out (146, 147).

Intriguingly, available data on derangements of the upstream hypothalamus/pituitary/gonadal axis showed an inconsistent association with TC. In particular, activating and inactivating mutations of the LR-receptor (LHR) gene are causative of DSD forms like isosexual precocious puberty in boys and Leydig cell hypoplasia, respectively. The latter presents variably from normal appearing female external genitalia to hypergonadotropic hypogonadism with microphallus and hypoplastic male external genitalia (148, 149). However, few available data documented TC only in patients with isosexual precocious puberty, particularly for testicular interstitial cell tumor observed in a 9 years old boy (with no available genetic screening at the time of the analysis), and two cases of activating mutations of the *LHR* gene reporting testicular seminoma in adult life (150–152).

## Testis Cancer: Something in Between Genes, Environment, and Hormones

As for the majority of oncological diseases, TC is the result of a complex interaction among the aforementioned genetic, environmental, and hormonal risk factors. To this regard, testicular dysgenesis syndrome (TDS) is probably the most reliable and clinically adherent model that describes the actual pathogenesis of TC (153).

The concept of TDS derives from the observation of the worldwide increasing incidence of a cluster of male urinary-genital alterations such as infertility, cryptorchidism, hypospadias and, indeed, TC. All these clinical conditions share the common feature of originating during fetal life, during which the impaired function of Sertoli and/or Leydig cells alters the proper testis function from germ line development to hormone production. The combination of these conditions results in a range of clinical consequences in adult age, spanning from infertility, to disorders of sexual development and cryptorchidism that, in turn, are risk factors for TC (154). In addition to the already mentioned genetic factors, there is a general agreement according to which the failure of testis nourishing cells is due to environmental factors, such as the exposure to chemical pollutants with endocrine disrupting activities (155). This hypothesis was originally suggested by the wide geographical variation observed for TDS symptoms in different countries. The most striking evidence, suggesting the possible influence of anthropogenic factors, was surely the differential prevalence of TDS-related disorders observed in two nations at equal latitude and industrialization, respectively, Denmark, with “higher” prevalence of TDS symptoms, and Finland with “lower” prevalence of TDS symptoms (156–159).

The mechanistic hypothesis of the endocrine disruption in TDS relies on two main pathways. The first one is the estrogen hypothesis, consisting of the exposure to chemicals with estrogenic properties that exert a central disruption of the hypothalamus/pituitary/gonadal axis, reducing in turn T release from the testis. Diethylstilbestrol (DES) is a clear example of an endocrine disruptor with estrogen action. Being an estrogen receptor agonist, DES was frequently prescribed to pregnant women during the 50s−60s in order to relieve abortions and pregnancy-related complications. However, males born from DES-treated mothers showed an increased incidence of epididymal cysts, altered sperm parameters, cryptorchidism, and TC (160–162). Similar to DES, the common plastic additive bisphenol A (BPA) was also acknowledged as a partial estrogen agonist (163). Exposure to BPA in males has been associated with increased levels of prolactin, estradiol, and the sex hormone-binding globulin level (164). Furthermore, higher levels of BPA in semen were associated with signs of a direct impairment of spermatogenesis such as poor sperm count and function (165).

Another suggested mechanism of endocrine disruption in males is the anti-androgen activity, typically exerted by phthalates plasticizers (166). Data obtained in cell and animal models are highly suggestive of direct influence of phthalates compounds on the endocrine function of Leydig cells, particularly impairing T and INSL3 production (167–169). Accordingly, increased prevalence of reduced anogenital distance (AGD), cryptorchidism, hypospadias, and other genital-urinary disorders were observed in male subjects from mothers exposed to this class of chemicals (170).

The list of substances with acknowledged or possible endocrine disrupting effects is continuously increasing and the exposure to these environmental agents is currently considered the major causative factor of the increasing incidence of TC throughout the last decades. However, equal exposure to the same disruptor is not univocally associated with the same phenotype of TDS, highlighting the role of the genetic background in the establishment of the susceptibility to genital-urinary disorders, in general, and to TC in particular (171). An interesting example of how genetic and environmental factors interact, determining a clinical phenotype, has been provided by our group in a very recent investigation focused on the *E2F1* gene (172). As cited above, altered *E2F1* expression has been significantly associated to several testis disorders such as spermatogenic impairment, cryptorchidism, and TC, particular in those cases of increased gene expression related to supernumerary gene copy numbers (49). Interestingly, through the use of an engineered NTERA-2 cl.D1 cell model, cultured at over-physiological temperature, the *E2F1* gene expression was up-regulated in a temperature- and gene-copy number- dependent manner. Altogether, these results suggest that the clinical condition associated with abnormal E2F1 expression, due to copy number variation, can be worsened even more by other concomitant environmental conditions, such as heat stress or an history of cryptorchidism, with a likely impact on TC development.

## Conclusions and Future Perspectives

TC is the most prevalent tumor disease in male subjects at reproductive age, showing a progressive increase throughout the last four decades. The current model that better explains this trend, based on clinical and experimental evidence, relies on the increased exposure to environmental factors, particularly chemical pollutants with endocrine disrupting activity, that alters the major hormonal axis that drives testis development and function from gestational age. The susceptibility to these alterations further depends on genetic factors that strongly justify the strong familiarity of TC (Table 1).

**Table 1:** Genetic factors and related mechanism associated to testis cancer.

**Gene**	**Mechanism**	**References**
*GATA4, GATA1*	Specification and differentiation of postnatal testicular development	(25–31)
*PRDM14, DMRT1*	Germ cell specification-sex determination	(25, 32–35)
*SALL4*	Disruption of POU5F1 binding motif; maintenance of pluripotency in embryonic stem cells	(25, 36)
*TEX14, WDR73, PMF1, CENPE, and PCNT*	Microtubule and chromosomal assembly; kinetochore-microtubule assembly; microtubule organization during interphase; microtubule assembly–related genes	(25, 37–44)
*KIT*	KIT–MAPK signaling	(25)
*FCP2L1, ZFP42*	Germ cell development and pluripotency	(45)
*ZWILCH*	Kinetochore function	(45)
*TIPIN*	Response to DNA damage	(45)
*TKTL1, LHPP*	Mitochondrial function	(45)
*E2F1*	Copy number variation	(49)
*INSL3, RXFP2*	Cryptorchidism	(55, 56, 63–65)
*AR, 17β-HSD*	Cryptorchidism; steroidogenesis; disorders of sex development	(55, 56, 63, 66–68, 133, 134, 137, 138)
*AMH, AMH type II receptor*	Cryptorchidism; disorders of sex development	(85, 86, 139–145)
*LHR*	Steroidogenesis	(150–152)

A very recent field of investigation that aims to integrate genetic and environmental factors on the risk for TC is epigenetics, namely the inheritance of genetic factors that do not rely on the variation of the genetic sequence but rather on the regulation of gene expression trough DNA methylation and histone modification. Very recent investigations showed that DNA from tumor cells display significant hypomethylation compared to normal germ cells, evidence likely due to the over-expression of de-methylating factors that are generally suppressed after fetal germ cell development (173). It is a shared opinion that environmental factors largely govern the balance between methylating and de-methylating factors (174).

Finally, it should be noted that in spite of the high sensitivity of TC to chemotherapy, explaining the good prognosis of treatment, there is still a large population of patients suffering from drug resistance or inefficient treatment settings among chemotherapy, radiotherapy, or surveillance (175). In this regard, some pioneering studies have succeeded in identifying genetic markers of good responses or tolerability to therapeutic agents, thus improving the overall outcome of treatments. This is the case for the identification of genetic variants in the *SLC16A5* gene, which has been significantly associated to ototoxicity induced by cisplatin (176).

In conclusion, the availability of novel strategies of investigation are of paramount importance to clarify the key aspects of TC development, progression and therapy, in order to further improve the prevention and treatment of a highly curable disease with an unexplained increasing diffusion.

## Author Contributions

AG conceived the manuscript. IC, MG, and IŠ performed bibliographic research. CF supervised manuscript draft. LD wrote most of the manuscript.

### Conflict of Interest Statement

The authors declare that the research was conducted in the absence of any commercial or financial relationships that could be construed as a potential conflict of interest.

## References

[1] Ferlay J Soerjomataram I Dikshit R Eser S Mathers C Rebelo M. Cancer incidence and mortality worldwide: sources, methods and major patterns in GLOBOCAN 2012. Int J Cancer. (2015) 136:E359–86. 10.1002/ijc.29210 25220842

[2] Ferlay J Soerjomataram I Ervik M Dikshit R Eser S Mathers C GLOBOCAN 2012 v1.0, Cancer Incidence and Mortality Worldwide: IARC Cancer Base No. 11. International Agency for Research on Cancer (2013). http://publications.iarc.fr/Databases/Iarc-Cancerbases/GLOBOCAN-2012-Estimated-Cancer-Incidence-Mortality-And-Prevalence-Worldwide-In-2012-V1.0-2012

[3] Hanna N Einhorn LH. Testicular cancer: a reflection on 50 years of discovery. J Clin Oncol. (2014) 32:3085–92. 10.1200/JCO.2014.56.0896 25024068

[4] Boccellino M Vanacore D Zappavigna S Cavaliere C Rossetti S D'Aniello C. Testicular cancer from diagnosis to epigenetic factors. Oncotarget. (2017) 8:104654–63. 10.18632/oncotarget.20992 29262668 PMC5732834

[5] Znaor A Lortet-Tieulent J Jemal A Bray F. International variations and trends in testicular cancer incidence and mortality. Eur Urol. (2014) 65:1095–106. 10.1016/j.eururo.2013.11.004 24268506

[6] Ghazarian AA Trabert B Devesa SS McGlynn KA. Recent trends in the incidence of testicular germ cell tumors in the United States. Andrology. (2015) 3:13–8. 10.1111/andr.288 25331158 PMC4342271

[7] Mortensen MS Lauritsen J Kier MG Bandak M Appelt AL Agerbæk M. Late relapses in stage I testicular cancer patients on surveillance. Eur Urol. (2016) 70:365–71. 10.1016/j.eururo.2016.03.016 26996661

[8] Warde PR Gospodarowicz MK Goodman PJ Sturgeon JF Jewett MA Catton CN. Results of a policy of surveillance in stage I testicular seminoma. Int J Radiat Oncol Biol Phys. (1993) 27:11–5. 10.1016/0360-3016(93)90415-R 8365931

[9] Aparicio J Maroto P García del Muro X Sánchez-Muñoz A Gumà J Margelí M. Prognostic factors for relapse in stage I seminoma: a new nomogram derived from three consecutive, risk-adapted studies from the Spanish Germ Cell Cancer Group (SGCCG). Ann Oncol. (2014) 25:2173–8. 10.1093/annonc/mdu437 25210015

[10] Aziz NM. Cancer survivorship research: state of knowledge, challenges and opportunities. Acta Oncol. (2007) 46:417–32. 10.1080/02841860701367878 17497308

[11] Ghezzi M Berretta M Bottacin A Palego P Sartini B Cosci I. Impact of bep or carboplatin chemotherapy on testicular function and sperm nucleus of subjects with testicular germ cell tumor. Front Pharmacol. (2016) 7:122. 10.3389/fphar.2016.00122 27242529 PMC4865517

[12] Ghezzi M De Toni L Palego P Menegazzo M Faggian E Berretta M. Increased risk of testis failure in testicular germ cell tumor survivors undergoing radiotherapy. Oncotarget. (2017) 9:3060–8. 10.18632/oncotarget.23081 29423028 PMC5790445

[13] Westergaard T Olsen JH Frisch M Kroman N Nielsen JW Melbye M. Cancer risk in fathers and brothers of testicular cancer patients in Denmark. A population-based study. Int J Cancer. (1996) 66:627–31. 10.1002/(SICI)1097-0215(19960529)66:5<627::AID-IJC8>3.3.CO;2-Y 8647624

[14] Gundy S Babosa M Baki M Bodrogi I. Increased predisposition to cancer in brothers and offspring of testicular tumor patients. Pathol Oncol Res. (2004) 10:197–203. 10.1007/BF03033760 15619639

[15] Hemminki K Chen B. Familial risks in testicular cancer as aetiological clues. Int J Androl. (2006) 29:205–10. 10.1111/j.1365-2605.2005.00599.x 16466541

[16] Czene K Lichtenstein P Hemminki K. Environmental and heritable causes of cancer among 9.6 million individuals in the Swedish Family-Cancer Database. Int J Cancer. (2002) 99:260–6. 10.1002/ijc.10332 11979442

[17] Mucci LA Hjelmborg JB Harris JR Czene K Havelick DJ Scheike T. Familial risk and heritability of cancer among twins in Nordic countries. JAMA. (1998) 315:68–76. 10.1001/jama.2015.17703 26746459 PMC5498110

[18] Crockford GP Linger R Hockley S Dudakia D Johnson L Huddart R. Genome-wide linkage screen for testicular germ cell tumour susceptibility loci. Hum Mol Genet. (2006) 15:443–51. 10.1093/hmg/ddi459 16407372

[19] Chung CC Kanetsky PA Wang Z Hildebrandt MA Koster R Skotheim RI. Meta-analysis identifies four new loci associated with testicular germ cell tumor. Nat Genet. (2013) 45:680–5. 10.1038/ng.2634 23666239 PMC3723930

[20] Kristiansen W Karlsson R Rounge TB Whitington T Andreassen BK Magnusson PK. Two new loci and gene sets related to sex determination and cancer progression are associated with susceptibility to testicular germ cell tumor. Hum Mol Genet. (2015) 24:4138–46. 10.1093/hmg/ddv129 25877299

[21] Ruark E Seal S McDonald H Zhang F Elliot A Lau K. Identification of nine new susceptibility loci for testicular cancer, including variants near DAZL and PRDM14. Nat Genet. (2013) 45:686–9. 10.1038/ng.2635 23666240 PMC3680037

[22] Dalgaard MD Weinhold N Edsgärd D Silver JD Pers TH Nielsen JE. A genome-wide association study of men with symptoms of testicular dysgenesis syndrome and its network biology interpretation. J Med Genet. (2012) 49:58–65. 10.1136/jmedgenet-2011-100174 22140272 PMC3284313

[23] Nathanson KL Kanetsky PA Hawes R Vaughn DJ Letrero R Tucker K. The Y deletion gr/gr and susceptibility to testicular germ cell tumor. Am J Hum Genet. (2005) 77:1034–43. 10.1086/498455 16380914 PMC1285161

[24] Litchfield K Summersgill B Yost S Sultana R Labreche K Dudakia D. Whole-exome sequencing reveals the mutational spectrum of testicular germ cell tumours. Nat Commun. (2015) 6:5973. 10.1038/ncomms6973 25609015 PMC4338546

[25] Litchfield K Levy M Orlando G Loveday C Law PJ Migliorini G. Identification of 19 new risk loci and potential regulatory mechanisms influencing susceptibility to testicular germ cell tumor. Nat Genet. (2017) 49:1133–40. 10.1038/ng.3896 28604728 PMC6016736

[26] Agnihotri S Wolf A Munoz DM Smith CJ Gajadhar A Restrepo A. A GATA4-regulated tumor suppressor network represses formation of malignant human astrocytomas. J Exp Med. (2011) 208:689–702. 10.1084/jem.20102099 21464220 PMC3135351

[27] Hellebrekers DM Lentjes MH van den Bosch SM Melotte V Wouters KA Daenen KL. GATA4 and GATA5 are potential tumor suppressors and biomarkers in colorectal cancer. Clin Cancer Res. (2009) 15:3990–7. 10.1158/1078-0432.CCR-09-0055 19509152

[28] Tsang AP Visvader JE Turner CA Fujiwara Y Yu C Weiss MJ. FOG, a multitype zinc finger protein, acts as a cofactor for transcription factor GATA-1 in erythroid and megakaryocytic differentiation. Cell. (1997) 90:109–19. 10.1016/S0092-8674(00)80318-9 9230307

[29] Adzhubei I Jordan DM Sunyaev SR. Predicting functional effect of human missense mutations using PolyPhen-2. Curr Protoc Hum Genet. (2013) Chapter 7:Unit 7.20. 10.1002/0471142905.hg0720s76 23315928 PMC4480630

[30] Ketola I Anttonen M Vaskivuo T Tapanainen JS Toppari J Heikinheimo M. Developmental expression and spermatogenic stage specificity of transcription factors GATA-1 and GATA-4 and their cofactors FOG-1 and FOG-2 in the mouse testis. Eur J Endocrinol. (2002) 147:397–406. 10.1530/eje.0.1470397 12213678

[31] Zheng R Blobel GA. GATA transcription factors and cancer. Genes Cancer. (2010) 1:1178–88. 10.1177/1947601911404223 21779441 PMC3092280

[32] Kurimoto K Yamaji M Seki Y Saitou M. Specification of the germ cell lineage in mice: a process orchestrated by the PR-domain proteins, Blimp1 and Prdm14. Cell Cycle. (2008) 7:3514–8. 10.4161/cc.7.22.6979 19001867

[33] Ohinata Y Ohta H Shigeta M Yamanaka K Wakayama T Saitou M. A signaling principle for the specification of the germ cell lineage in mice. Cell. (2009) 137:571–84. 10.1016/j.cell.2009.03.014 19410550

[34] Yamaji M Seki Y Kurimoto K Yabuta Y Yuasa M Shigeta M. Critical function of Prdm14 for the establishment of the germ cell lineage in mice. Nat Genet. (2008) 40:1016–22. 10.1038/ng.186 18622394

[35] Smith CA McClive PJ Western PS Reed KJ Sinclair AH. Conservation of a sex-determining gene. Nature. (1999) 402:601–2. 10.1038/45130 10604464

[36] Rao S Zhen S Roumiantsev S McDonald LT Yuan GC Orkin SH. Differential roles of Sall4 isoforms in embryonic stem cell pluripotency. Mol Cell Biol. (2010) 30:5364–80. 10.1128/MCB.00419-10 20837710 PMC2976381

[37] Bojesen SE Pooley KA Johnatty SE Beesley J Michailidou K Tyrer JP. Multiple independent variants at the TERT locus are associated with telomere length and risks of breast and ovarian cancer. Nat Genet. (2013) 45:371–84. 10.1038/ng.2566 23535731 PMC3670748

[38] Mondal G Ohashi A Yang L Rowley M Couch FJ. Tex14, a Plk1-regulated protein, is required for kinetochore–microtubule attachment and regulation of the spindle assembly checkpoint. Mol Cell. (2012) 45:680–95. 10.1016/j.molcel.2012.01.013 22405274 PMC3302152

[39] Jinks RN Puffenberger EG Baple E Harding B Crino P Fogo AB. Recessive nephrocerebellar syndrome on the Galloway–Mowat syndrome spectrum is caused by homozygous protein-truncating mutations of WDR73. Brain. (2015) 138:2173–90. 10.1093/brain/awv153 26070982 PMC4511861

[40] Colin E Huynh Cong E Mollet G Guichet A Gribouval O Arrondel C. Loss-of-function mutations in WDR73 are responsible for microcephaly and steroid-resistant nephrotic syndrome: Galloway–Mowat syndrome. Am J Hum Genet. (2014) 95:637–48. 10.1016/j.ajhg.2014.10.011 25466283 PMC4259970

[41] Petrovic A Pasqualato S Dube P Krenn V Santaguida S Cittaro D. The MIS12 complex is a protein interaction hub for outer kinetochore assembly. J Cell Biol. (2010) 190:835–52. 10.1083/jcb.201002070 20819937 PMC2935574

[42] Rao CV Yamada HY Yao Y Dai W. Enhanced genomic instabilities caused by deregulated microtubule dynamics and chromosome segregation: a perspective from genetic studies in mice. Carcinogenesis. (2009) 30:1469–74. 10.1093/carcin/bgp081 19372138 PMC2736299

[43] Barisic M Silva e Sousa R Tripathy SK Magiera MM Zaytsev AV Pereira AL. Microtubule detyrosination guides chromosomes during mitosis. Science. (2015) 348:799–803. 10.1126/science.aaa5175 25908662 PMC4506776

[44] Ma W Viveiros MM. Depletion of pericentrin in mouse oocytes disrupts microtubule organizing center function and meiotic spindle organization. Mol Reprod Dev. (2014) 81:1019–29. 10.1002/mrd.22422 25266793 PMC4229429

[45] Wang Z McGlynn KA Rajpert-De Meyts E Bishop DT Chung CC Dalgaard MD. Meta-analysis of five genome-wide association studies identifies multiple new loci associated with testicular germ cell tumor. Nat Genet. (2017) 49:1141–7. 10.1038/ng.3879 28604732 PMC5490654

[46] de Smith AJ Walters RG Froguel P Blakemore AI. Human genes involved in copy number variation: mechanisms of origin, functional effects and implications for disease. Cytogenet Genome Res. (2008) 123:17–26. 10.1159/000184688 19287135 PMC2920180

[47] Stadler ZK Esposito D Shah S Vijai J Yamrom B Levy D. Rare *de novo* germline copy-number variation in testicular cancer. Am J Hum Gen. (2012) 91:379–83. 10.1016/j.ajhg.2012.06.019 22863192 PMC3415553

[48] Edsgärd D Dalgaard MD Weinhold N Wesolowska-Andersen A Rajpert-De Meyts E Ottesen AM. Genome-wide assessment of the association of rare and common copy number variations to testicular germ cell cancer. Front Endocrinol. (2013) 4:2. 10.3389/fendo.2013.00002 23372565 PMC3557397

[49] Rocca MS Di Nisio A Marchiori A Ghezzi M Opocher G Foresta C. Copy number variations of E2F1: a new genetic risk factor for testicular cancer. Endocr Relat Cancer. (2017) 24:119–25. 10.1530/ERC-16-0514 28104681

[50] Sengupta S Henry RW. Regulation of the retinoblastoma-E2F pathway by the ubiquitin-proteasome system. Biochim Biophys Acta. (2015) 1849:1289–97. 10.1016/j.bbagrm.2015.08.008 26319102

[51] Johnson DG. The paradox of E2F1: oncogene and tumor suppressor gene. Mol Carcinog. (2000) 27:151–7. 10.1002/(SICI)1098-2744(200003)27:3<151::AID-MC1>3.0.CO;2-C 10708476

[52] Bertoli C Skotheim JM de Bruin RA. Control of cell cycle transcription during G1 and S phases. Nat Rev Mol Cell Biol. (2013) 14:518–28. 10.1038/nrm3629 23877564 PMC4569015

[53] Giacinti C Giordano A. RB and cell cycle progression. Oncogene. (2006) 25:5220–7. 10.1038/sj.onc.1209615 16936740

[54] Ladu S Calvisi DF Conner EA Farina M Factor VM Thorgeirsson SS. E2F1 inhibits c-Myc-driven apoptosis via PIK3CA/Akt/mTOR and COX-2 in a mouse model of human liver cancer. Gastroenterology. (2008) 135:1322–32. 10.1053/j.gastro.2008.07.012 18722373 PMC2614075

[55] Purdue MP Devesa SS Sigurdson AJ McGlynn KA. International patterns and trends in testis cancer incidence. Int J Cancer. (2005) 115:822–7. 10.1002/ijc.20931 15704170

[56] Bray F Richiardi L Ekbom A Pukkala E Cuninkova M Møller H. Trends in testicular cancer incidence and mortality in 22 European countries: continuing increases in incidence and declines in mortality. Int J Cancer. (2006) 118:3099–111. 10.1002/ijc.21747 16395710

[57] Hutson JM Li R Southwell BR Newgreen D Cousinery M Regulation of testicular descent. Pediatr Surg Int. (2015) 31:317–25. 10.1007/s00383-015-3673-4 25690562

[58] Marin P Ferlin A Moro E Rossi A Bartoloni L Rossato M. Novel insulin-like 3 (INSL3) gene mutation associated with human cryptorchidism. Am J Med Genet. (2001) 103:348–9. 10.1002/ajmg.1579 11746019

[59] Kumagai J Hsu SY Matsumi H Roh JS Fu P Wade JD. INSL3/Leydig insulin-like peptide activates the LGR8 receptor important in testis descent. J Biol Chem. (2002) 277:31283–6. 10.1074/jbc.C200398200 12114498

[60] Ivell R Hartung S. The molecular basis of cryptorchidism. Mol Hum Reprod. (2003) 9:175–81. 10.1093/molehr/gag025 12651898

[61] Tanyel FC Ertunç M Ekinci S Yildirim M Onur R. Anti-androgen induced inhibition of testicular descent is associated with a decrease in sympathetic tonus. Eur J Pediatr Surg. (2005) 15:273–8. 10.1055/s-2005-837625 16163594

[62] Nation TR Buraundi S Balic A Farmer PJ Newgreen D Southwell BR. The effect of flutamide on expression of androgen and estrogen receptors in the gubernaculum and surrounding structures during testicular descent. J Pediatr Surg. (2011) 46:2358–62. 10.1016/j.jpedsurg.2011.09.026 22152882

[63] Ferlin A Zuccarello D Zuccarello B Chirico MR Zanon GF Foresta C. Genetic alterations associated with cryptorchidism. JAMA. (2008) 300:2271–6. 10.1001/jama.2008.668 19017913

[64] Krausz C Quintana-Murci L Fellous M Siffroi JP McElreavey K. Absence of mutations involving the INSL3 gene in human idiopathic cryptorchidism. Mol Hum Reprod. (2000) 6:298–302. 10.1093/molehr/6.4.298 10729310

[65] Mamoulakis C Georgiou I Dimitriadis F Tsounapi P Giannakis I Chatzikyriakidou A. Genetic analysis of the human insulin-like 3 gene: absence of mutations in a Greek paediatric cohort with testicular maldescent. Andrologia. (2014) 46:986–96. 10.1111/and.12184 25728210

[66] Wiener JS Marcelli M Gonzales ET Roth DR Lamb DJ Androgen receptor gene alterations are not associated with isolated cryptorchidism. J. Urol. (1998) 160:863–5. 10.1097/00005392-199809010-00079 9720578

[67] Radpour R Rezaee M Tavasoly A Solati S Saleki A. Association of long polyglycine tracts (GGN repeats) in exon 1 of the androgen receptor gene with cryptorchidism and penile hypospadias in Iranian patients. J Androl. (2007) 28:164–9. 10.2164/jandrol.106.000927 16957138

[68] Ferlin A Garolla A Bettella A Bartoloni L Vinanzi C Roverato A. Androgen receptor gene CAG and GGC repeat lengths in cryptorchidism. Eur J Endocrinol. (2005) 152:419–25. 10.1530/eje.1.01860 15757859

[69] Bojesen A Juul S Birkebaek NH Gravholt CH. Morbidity in Klinefelter syndrome: a Danish register study based on hospital discharge diagnoses. J Clin Endocrinol Metab. (2006) 91:1254–60. 10.1210/jc.2005-0697 16394093

[70] Accardo G Vallone G Esposito D Barbato F Renzullo A Conzo G. Testicular parenchymal abnormalities in Klinefelter syndrome: a question of cancer? Examination of 40 consecutive patients. Asian J Androl. (2015) 17:154–8. 10.4103/1008-682X.128514 25130577 PMC4291860

[71] Carroll PR Morse MJ Koduru PP Chaganti RS. Testicular germ cell tumor in patient with Klinefelter syndrome. Urology. (1988) 31:72–4. 10.1016/0090-4295(88)90579-1 3336933

[72] Ferguson-Smith MA. The prepubertal testicular lesion in chromatin-positive Klinefelter's syndrome (primary micro-orchidism) as seen in mentally handicapped children. Lancet. (1959) 1:219–22. 10.1016/S0140-6736(59)90049-2 13631972

[73] Fishman MD Eisenberg DA Horrow MM. Klinefelter syndrome with leydig cell tumor/hyperplasia. Ultrasound Q. (2010) 26:101–2. 10.1097/RUQ.0b013e3181dd27d0 20498565

[74] Gustavson KH Gamstorp I Meurling S. Bilateral teratoma of testis in two brothers with 47,XXY Klinefelter's syndrome. Clin Genet. (1975) 8:5–10. 10.1111/j.1399-0004.1975.tb01947.x 1149322

[75] Hasle H Mellemgaard A Nielsen J Hansen J. Cancer incidence in men with Klinefelter syndrome. Br J Cancer. (1995) 71:416–20. 10.1038/bjc.1995.85 7841064 PMC2033611

[76] Isurugi K Imao S Hirose K Aoki H. Seminoma in Klinefelter's syndrome with 47, XXY, 15s^+^ karyotype. Cancer. (1977) 39:2041–7. 10.1002/1097-0142(197705)39:5<2041::AID-CNCR2820390521>3.0.CO;2-X 858129

[77] Maqdasy S Bogenmann L Batisse-Lignier M Roche B Franck F Desbiez F. Leydig cell tumor in a patient with 49,XXXXY karyotype: a review of literature. Reprod Biol Endocrinol. (2015) 13:72. 10.1186/s12958-015-0071-7 26160035 PMC4496935

[78] Poster RB Katz DS. Leydig cell tumor of the testis in Klinefelter syndrome: MR detection. J Comput Assist Tomogr. (1993) 17:480–1. 10.1097/00004728-199305000-00028 8491916

[79] Pradhan D Kaman L Dhillon J Mohanty SK. Mediastinal mixed germ cell tumor in an infertile male with Klinefelter syndrome: a case report and literature review. J Cancer Res Ther. (2015) 11:1034. 10.4103/0973-1482.150697 26881632

[80] Reddy SR Svec F Richardson P. Seminoma of the testis in a patient with 48,XXYY variant of Klinefelter's syndrome. South Med J. (1991) 84:773–5. 10.1097/00007611-199106000-00026 2052972

[81] Shaw NM Stauffer C Eisenberg ML. Leydig cell tumor found incidentally during microscopic testicular sperm extraction in patient with mosaic Klinefelter syndrome: case report. Fertil Steril. (2016) 106:1344–7. 10.1016/j.fertnstert.2016.07.1116 27523297

[82] Simpson JL Photopulos G. Letter: bilateral teratoma of testis in 2 brothers with 47,XXY Klinefelter's syndrome. Clin Genet. (1976) 9:380–1. 1261078

[83] Soria JC Durdux C Chrétien Y Sibony M Damotte D Housset M. Malignant Leydig cell tumor of the testis associated with Klinefelter's syndrome. Anticancer Res. (1999) 19:4491–4. 10650798

[84] Stevens MJ Jameson CF Hendry WF. Bilateral testicular teratoma in Klinefelter's syndrome. Br J Urol. (1993) 72:384–5. 10.1111/j.1464-410X.1993.tb00743.x 8221006

[85] Abduljabbar M Taheini K Picard JY Cate RL Josso N. Mutations of the AMH type II receptor in two extended families with persistent Mullerian duct syndrome: lack of phenotype/genotype correlation. Horm Res Paediatr. (2008) 77:291–7. 10.1159/000338343 22584735

[86] Josso N Picard JY Imbeaud S Carré ED Zeller J Adamsbaum C. The persistent müllerian duct syndrome: a rare cause of cryptorchidism. Eur J Pediatr. (1993) 152: S76–8. 10.1007/BF02125444 8101816

[87] Lymperi S Giwercman A. Endocrine disruptors and testicular function. Metab Clin Exp. (2018) 86:79–90. 10.1016/j.metabol.2018.03.022 29605435

[88] Stoll C Alembik Y Roth MP Dott B. Genetic and environmental factors in hypospadias. J Med Genet. (1990) 27:559–63. 10.1136/jmg.27.9.559 2231648 PMC1017217

[89] Schnack TH Zdravkovic S Myrup C Westergaard T Wohlfahrt J Melbye M. Familial aggregation of cryptorchidism—a nationwide cohort study. Am J Epidemiol. (2008) 167:1453–7. 10.1093/aje/kwn081 18436537

[90] McGlynn KA Trabert B. Adolescent and adult risk factors for testicular cancer. Nat Rev Urol. (2012) 9:339–49. 10.1038/nrurol.2012.61 22508459 PMC4031676

[91] Parkin DM. The global health burden of infection-associated cancers in the year 2002. Int J Cancer. (2006) 118:3030–44. 10.1002/ijc.21731 16404738

[92] Algood CB Newell GR Johnson DE. Viral etiology of testicular tumors. J Urol. (1988) 139:308–10. 10.1016/S0022-5347(17)42394-9 2828695

[93] Rajpert-De Meyts E Hørding U Nielsen HW Skakkebaek NE. Human papillomavirus and Epstein-Barr virus in the etiology of testicular germ cell tumours. APMIS. (1994) 102:38–42. 10.1111/j.1699-0463.1994.tb04842.x 8166998

[94] Yousif L Hammer GP Blettner M Zeeb H. Testicular cancer and viral infections: a systematic literature review and meta-analysis. J Med Virol. (2013) 85:2165–75. 10.1002/jmv.23704 23959966

[95] Hentrich M Pfister D. HIV-associated urogenital malignancies. Oncol Res Treat. (2017) 40:106–12. 10.1159/000457130 28253510

[96] Aminsharifi A Monsef A Noorafshan A Karbalay-Doust S Jafarinezhad Z Koohi-Hosseinabadi O. Effects of intratesticular hematoma on testis microstructure, spermatogenesis, and testosterone production: defining a cutoff point for significant intratesticular hematoma. Urology. (2018) 118:80–6. 10.1016/j.urology.2018.05.005 29777790

[97] Dusek L Abrahamova J Lakomy R Vyzula R Koptikova J Pavlik T. Multivariate analysis of risk factors for testicular cancer: a hospital-based case-control study in the Czech Republic. Neoplasma. (2008) 55:356–68. Available online at: https://www.ncbi.nlm.nih.gov/pubmed/?term=Dusek+L%2C+Abrahamova+J%2C+Lakomy+R%2C+Vyzula+R%2C+Koptikova+J%2C+Pavlik+T%2C+Muzik+J%2C+Klimes+D 18505349

[98] Sandella B Hartmann B Berkson D Hong E. Testicular conditions in athletes: torsion, tumors, and epididymitis. Curr Sports Med Rep. (2012) 11:92–5. 10.1249/JSR.0b013e31824c8886 22410701

[99] Coldman AJ Elwood JM Gallagher RP. Sports activities and risk of testicular cancer. Br J Cancer. (1982) 46:749–56. 10.1038/bjc.1982.267 6128995 PMC2011166

[100] Haughey BP Graham S Brasure J Zielezny M Sufrin G Burnett WS. The epidemiology of testicular cancer in upstate New York. Am J Epidemiol. (1989) 130:25–36. 10.1093/oxfordjournals.aje.a115319 2568087

[101] Littman AJ Doody DR Biggs ML Weiss NS Starr JR Schwartz SM. Physical activity in adolescence and testicular germ cell cancer risk. Cancer Causes Control. (2009) 20:1281–90. 10.1007/s10552-009-9347-6 19399630 PMC2890221

[102] Kim MK Zoh KD. Fate and transport of mercury in environmental media and human exposure. J Prev Med Public Health. (2012) 45:335–43. 10.3961/jpmph.2012.45.6.335 23230463 PMC3514463

[103] de Angelis C Galdiero M Pivonello C Salzano C Gianfrilli D Piscitelli P. The environment and male reproduction: the effect of cadmium exposure on reproductive function and its implication in fertility. Reprod Toxicol. (2017) 73:105–27. 10.1016/j.reprotox.2017.07.021 28774687

[104] Rana SV. Perspectives in endocrine toxicity of heavy metals–a review. Biol Trace Elem Res. (2014) 160:1–14. 10.1007/s12011-014-0023-7 24898714

[105] Anderson MB Lepak K Farinas V George WJ. Protective action of zinc against cobalt-induced testicular damage in the mouse. Reprod. Toxicol. (1993) 7:49–54. 10.1016/0890-6238(93)90009-V 8448416

[106] Verhoeven RH Louwman MW Buntinx F Botterweck AM Lousbergh D Faes C. Variation in cancer incidence in northeastern Belgium and southeastern Netherlands seems unrelated to cadmium emission of zinc smelters. Eur J Cancer Prev. (2011) 20:549–55. 10.1097/CEJ.0b013e3283498e9c 21857522

[107] Nawrot T Plusquin M Hogervorst J Roels HA Celis H Thijs L. Environmental exposure to cadmium and risk of cancer: a prospective population-based study. Lancet Oncol. (2006) 7:119–26. 10.1016/S1470-2045(06)70545-9 16455475

[108] Rhomberg W Schmoll HJ Schneider B. High frequency of metalworkers among patients with seminomatous tumors of the testis: a case-control study. Am J Ind Med. (1995) 28:79–87. 10.1002/ajim.4700280107 7573077

[109] Hobbesland A Kjuus H Thelle DS. Study of cancer incidence among 8530 male workers in eight Norwegian plants producing ferrosilicon and silicon metal. Occup Environ Med. (1999) 56:625–31. 10.1136/oem.56.9.625 10615296 PMC1757784

[110] Mills PK Newell GR Johnson DE. Testicular cancer associated with employment in agriculture and oil and natural gas extraction. Lancet. (1984) 1:207–10. 10.1016/S0140-6736(84)92125-1 6141346

[111] McDowall ME Balarajan R Testicular cancer mortality in England and Wales 1971–1980: variations by occupation. J Epidemiol Community Health. (1986) 40:26–9. 10.1136/jech.40.1.26 3711767 PMC1052484

[112] Mills PK Newell GR. Testicular cancer risk in agricultural occupations. J Occup Med. (1984) 26:798–9. 10.1097/00043764-198411000-00003 6502282

[113] Blair A Zahm SH Pearce NE Heineman EF Fraumeni JF Jr. Clues to cancer etiology from studies of farmers. Scand J Work Environ Health. (1992) 18:209–15. 10.5271/sjweh.1578 1411362

[114] Acquavella J. Cancer among farmers: a meta-analysis. Ann Epidemiol. (1998) 8:64–74. 10.1016/S1047-2797(97)00120-8 9465996

[115] Toppari J. Environmental endocrine disrupters. Sex Dev. (2008) 2:260–7. 10.1159/000152042 18987500

[116] Abou-Donia MB Suliman HB Khan WA Abdel-Rahman AA. Testicular germ-cell apoptosis in stressed rats following combined exposure to pyridostigmine bromide, N,N-diethyl m-toluamide (DEET), and permethrin. J Toxicol Environ Health A. (2003) 66:57–73. 10.1080/15287390306463 12587291

[117] Ivell R. Lifestyle impact and the biology of the human scrotum. Reprod Biol Endocrinol. (2007) 5:15. 10.1186/1477-7827-5-15 17448228 PMC1863418

[118] Garolla A Torino M Sartini B Cosci I Patassini C Carraro U. Seminal and molecular evidence that sauna exposure affects human spermatogenesis. Hum Reprod. (2013) 28:877–85. 10.1093/humrep/det020 23411620

[119] Garolla A Šabović I Tescari S De Toni L Menegazzo M Cosci I. Impaired sperm function in infertile men relies on the membrane sterol pattern. Andrology. (2018) 6:325–34. 10.1111/andr.12468 29378089

[120] Zhang ZF Vena JE Zielezny M Graham S Haughey BP Brasure J. Occupational exposure to extreme temperature and risk of testicular cancer. Arch Environ Health. (1995) 50:13–8. 10.1080/00039896.1995.9955007 7717764

[121] Bates MN Fawcett J Garrett N Arnold R Pearce N Woodward A. Is testicular cancer an occupational disease of fire fighters? Am J Ind Med. (2001) 40:263–70. 10.1002/ajim.1097 11598972

[122] Karagas MR Weiss NS Strader CH Daling JR. Elevated intrascrotal temperature and the incidence of testicular cancer in noncryptorchid men. Am J Epidemiol. (1989) 129:1104–9. 10.1093/oxfordjournals.aje.a115232 2543215

[123] Fink G. 60 YEARS OF NEUROENDOCRINOLOGY: MEMOIR: Harris' neuroendocrine revolution: of portal vessels and self-priming. J Endocrinol. (2015) 226:T13–24. 10.1530/JOE-15-0130 25967698

[124] Fuxjager MJ Schuppe ER. Androgenic signaling systems and their role in behavioral evolution. J Steroid Biochem Mol Biol. (2018) 184:47–56. 10.1016/j.jsbmb.2018.06.004 29883693

[125] Rajpert-de Meyts E Hoei-Hansen CE. From gonocytes to testicular cancer: the role of impaired gonadal development. Ann NY Acad Sci. (2007) 1120:168–80. 10.1196/annals.1411.013 18184914

[126] Sperger JM Chen X Draper JS Antosiewicz JE Chon CH Jones SB. Gene expression patterns in human embryonic stem cells and human pluripotent germ cell tumors. Proc Natl Acad Sci USA. (2003) 100:13350–5. 10.1073/pnas.2235735100 14595015 PMC263817

[127] Gidekel S Pizov G Bergman Y Pikarsky E. Oct-3/4 is a dose-dependent oncogenic fate determinant. Cancer Cell. (2003) 4:361–70. 10.1016/S1535-6108(03)00270-8 14667503

[128] Almstrup K Hoei-Hansen CE Wirkner U Blake J Schwager C Ansorge W. Embryonic stem cell-like features of testicular carcinoma *in situ* revealed by genome-wide gene expression profiling. Cancer Res. (2004) 64:4736–43. 10.1158/0008-5472.CAN-04-0679 15256440

[129] Skotheim RI Lind GE Monni O Nesland JM Abeler VM Fosså SD. Differentiation of human embryonal carcinomas *in vitro* and *in vivo* reveals expression profiles relevant to normal development. Cancer Res. (2005) 65:5588–98. 10.1158/0008-5472.CAN-05-0153 15994931

[130] Rajpert-De Meyts E Hanstein R Jørgensen N Graem N Vogt PH Skakkebaek NE. Developmental expression of POU5F1 (OCT-3/4) in normal and dysgenetic human gonads. Hum Reprod. (2004) 19:1338–44. 10.1093/humrep/deh265 15105401

[131] Bowles J Knight D Smith C Wilhelm D Richman J Mamiya S. Retinoid signaling determines germ cell fate in mice. Science. (2006) 312:596–600. 10.1126/science.1125691 16574820

[132] Tadokoro-Cuccaro R Hughes IA. Androgen insensitivity syndrome. Curr Opin Endocrinol Diabetes Obes. (2014) 21:499–503. 10.1097/MED.0000000000000107 25354046

[133] Deeb A Mason C Lee YS Hughes IA. Correlation between genotype, phenotype and sex of rearing in 111 patients with partial androgen insensitivity syndrome. Clin Endocrinol. (2005) 63:56–62. 10.1111/j.1365-2265.2005.02298.x 15963062

[134] Jaaskelainen J. Molecular biology of androgen insensitivity. Mol Cell Endocrinol. (2012) 352:4–12. 10.1016/j.mce.2011.08.006 21871529

[135] MacLaughlin DT Donahoe PK. Sex determination and differentiation. N Engl J Med. (2004) 350:367–78. 10.1056/NEJMra022784 14736929

[136] Wuerstle M Lesser T Hurwitz R Applebaum H Lee SL. Persistent mullerian duct syndrome and transverse testicular ectopia: embryology, presentation, and management. J Pediatr Surg. (2007) 42:2116–9. 10.1016/j.jpedsurg.2007.09.003 18082721

[137] Cools M van Aerde K Kersemaekers AM Boter M Drop SL Wolffenbuttel KP. Morphological and immunohistochemical differences between gonadal maturation delay and early germ cell neoplasia in patients with undervirilization syndromes. J Clin Endocrinol Metab. (2005) 90:5295–303. 10.1210/jc.2005-0139 15998778

[138] Cools M Drop SL Wolffenbuttel KP Oosterhuis JW Looijenga LH. Germ cell tumors in the intersex gonad: old paths, new directions, moving frontiers. Endocr Rev. (2006) 27:468–84. 10.1210/er.2006-0005 16735607

[139] Giri SK Berney D O'Driscoll J Drumm J Flood HD Gupta RK. Choriocarcinoma with teratoma arising from an intra-abdominal testis in patient with persistent Müllerian duct syndrome. Lancet Oncol. (2004) 5:451–2. 10.1016/S1470-2045(04)01513-X 15231252

[140] Ramanujam AS Chandra A Raman SG Sagar TG Mallikarjuna VS. Persistent Müllerian Duct Syndrome (PMDS) with testicular seminoma. Indian J Pathol Microbiol. (2001) 44:441–3. 12035359

[141] Asthana S Deo SV Shukla NK Raina V Kumar L. Persistent Mullerian duct syndrome presenting with bilateral intra-abdominal gonadal tumours and obstructive uropathy. Clin Oncol. (2001) 13:304–6. 10.1007/s001740170061 11554632

[142] Dueñas A Saldívar C Castillero C Flores G Martínez P Jiménez M. A case of bilateral seminoma in the setting of persistent mullerian duct syndrome. Rev Invest Clin. (2001) 53:193–6. 11421115

[143] Williams JC Merguerian PA Schned AR Amdur RJ. Bilateral testicular carcinoma *in situ* in persistent müllerian duct syndrome: a case report and literature review. Urology. (1994) 44:595–8. 10.1016/S0090-4295(94)80068-5 7941204

[144] van Laarhoven CJ Juttmann JR Pijpers PM Roukema JA. A testicular tumour in the left adnex. The persistent mullerian duct syndrome with testicular malignancy. Eur J Surg Oncol. (1991) 17:97–8. 1671659

[145] Snow BW Rowland RG Seal GM Williams SD. Testicular tumor in patient with persistent müllerian duct syndrome. Urology. (1985) 26:495–7. 10.1016/0090-4295(85)90164-5 2865841

[146] Beatty JS Bhalla VK Hatley RM Pipkin WL Howell CG. Neglected cryptorchidism: delayed recognition of persistent müllerian duct syndrome and subsequent malignant degeneration. Urology. (2013) 82:511–4. 10.1016/j.urology.2013.05.020 23876582

[147] Lottrup G Jørgensen A Nielsen JE Jørgensen N Duno M Vinggaard AM. Identification of a novel androgen receptor mutation in a family with multiple components compatible with the testicular dysgenesis syndrome. J Clin Endocrinol Metab. (2013) 98:2223–9. 10.1210/jc.2013-1278 23589523

[148] Cutler GB Jr Overview of premature sexual development. In: Grave GD Cutler GB Jr editors. Sexual Precocity: Etiology, Diagnosis, and Management. New York, NY: Raven Press (1993). p. 1–10.

[149] Laue L Wu SM Kudo M Hsueh AJW Griffin JE Wilson JD The Gene defect that causes genetically XY males to develop as apparent females (Leydig cell hypoplasia). In: Selected Biomedical Research Summaries, 1994 ASCB Annual Meeting, Third Annual Press Book. MD: American Society for Cell Biology (1994).

[150] Röttger J Hadden DR Morrison E McKeown F. Isosexual precocious puberty in a 9-year-old boy: nodular interstitial cell hyperplasia. J Royal Soc Med. (1981) 74:66–8. 10.1177/014107688107400113 7463420 PMC1438318

[151] Martin MM Wu SM Martin AL Rennert OM Chan WY. Testicular seminoma in a patient with a constitutively activating mutation of the luteinizing hormone/chorionic gonadotropin receptor. Euro J Endocrinol. (1998) 139:101–6. 10.1530/eje.0.1390101 9703386

[152] Liu G Duranteau L Monroe J Doyle DA Carel JC Shenker A A novel somatic mutation of the lutropin receptor (LHR) gene in Leydig cell adenoma. Program and Abstracts. In: The 80th Annual Meeting of the Endocrine Society. (1998). 62 p.

[153] Juul A Almstrup K Andersson AM Jensen TK Jørgensen N Main KM. Possible fetal determinants of male infertility. Nat Rev Endocrinol. (2014) 10:553–62. 10.1038/nrendo.2014.97 24935122

[154] Rajpert-De Meyts E. Developmental model for the pathogenesis of testicular carcinoma *in situ*: genetic and environmental aspects. Hum Reprod Update. (2006) 12:303–23. 10.1093/humupd/dmk006 16540528

[155] Cevasco R Urbatzka S Bottero A Massari F Pedemonte W Kloas A. Endocrine disrupting chemicals (EDC) with (anti)estrogenic and (anti)androgenic modes of action affecting reproductive biology of Xenopus laevis: II. Effects on gonad histomorphology. Comp Biochem Physiol C Toxicol Pharmacol. (2008) 147:241–51. 10.1016/j.cbpc.2007.10.001 18032117

[156] Jensen TK Vierula M Hjollund NH Saaranen M Scheike T Saarikoski S. Semen quality among Danish and Finnish men attempting to conceive. The Danish first pregnancy planner study team. Eur J Endocrinol. (2000) 142:47–52. 10.1530/eje.0.1420047 10633221

[157] Boisen KA Kaleva M Main KM Virtanen HE Haavisto AM Schmidt IM. Difference in prevalence of congenital cryptorchidism in infants between two Nordic countries. Lancet. (2004) 363:1264–9. 10.1016/S0140-6736(04)15998-9 15094270

[158] Boisen KA Chellakooty M Schmidt IM Kai CM Damgaard IN Suomi AM. Hypospadias in a cohort of 1072 Danish newborn boys: prevalence and relationship to placental weight, anthropometrical measurements at birth, and reproductive hormone levels at three months of age. J Clin Endocrinol Metab. (2005) 90:4041–6. 10.1210/jc.2005-0302 15870122

[159] Richiardi L Bellocco R Adami HO Torrång A Barlow L Hakulinen T. Testicular cancer incidence in eight northern European countries: secular and recent trends. Cancer Epidemiol. Biomark Prev. (2004) 13:2157–66. Available online at: http://cebp.aacrjournals.org/content/cebp/13/12/2157.full.pdf 15598775

[160] Troisi R Hyer M Hatch EE Titus-Ernstoff L Palmer JR Strohsnitter WC. Medical conditions among adult offspring prenatally exposed to diethylstilbestrol. Epidemiology. (2013) 24:430–8. 10.1097/EDE.0b013e318289bdf7 23474687

[161] Martin OV Shialis T Lester JN Scrimshaw MD Boobis AR Voulvoulis N. Testicular dysgenesis syndrome and the estrogen hypothesis: a quantitative meta-analysis. Environ Health Perspect. (2008) 116:149–57. 10.1289/ehp.10545 18288311 PMC2235228

[162] Strohsnitter WC Noller KL Hoover RN Robboy SJ Palmer JR Titus-Ernstoff L. Cancer risk in men exposed *in utero* to diethylstilbestrol. J Natl Cancer Inst. (2001) 93:545–51. 10.1093/jnci/93.7.545 11287449

[163] Cabaton NJ Canlet C Wadia PR Tremblay-Franco M Gautier R Molina J. Effects of low doses of bisphenol a on the metabolome of perinatally exposed CD-1 mice. Environ Health Perspect. (2013) 121:586–93. 10.1289/ehp.1205588 23425943 PMC3673190

[164] Liu X Miao M Zhou Z Gao E Chen J Wang J. Exposure to bisphenol-A and reproductive hormones among male adults. Environ Toxicol Pharmacol. (2015) 39:934–41. 10.1016/j.etap.2015.03.007 25818109

[165] Vitku J Sosvorova L Chlupacova T Hampl R Hill M Sobotka V. Differences in bisphenol A and estrogen levels in the plasma and seminal plasma of men with different degrees of infertility. Physiol Res. (2015) 64:S303–11. Available online at: http://www.biomed.cas.cz/physiolres/pdf/64%20Suppl%202/64_S303.pdf 26680493 10.33549/physiolres.933090

[166] Blount BC Silva MJ Caudill SP Needham LL Pirkle JL Sampson EJ. Levels of seven urinary phthalate metabolites in a human reference population. Environ Health Perspect. (2000) 108:979–82. 10.1289/ehp.00108979 11049818 PMC1240132

[167] Gray LE Jr. Ostby J Furr J Price M Veeramachaneni DN Parks L. Perinatal exposure to the phthalates DEHP, BBP, and DINP, but not DEP, DMP, or DOTP, alters sexual differentiation of the male rat. Toxicol Sci. (2000) 58:350–65. 10.1093/toxsci/58.2.350 11099647

[168] Zhou D Wang H Zhang J. Di-n-butyl phthalate (DBP) exposure induces oxidative stress in epididymis of adult rats. Toxicol Ind Health. (2011) 27:65–71. 10.1177/0748233710381895 20823052

[169] Hu Y Dong C Chen M Lu J Han X Qiu L. Low-dose monobutyl phthalate stimulates steroidogenesis through steroidogenic acute regulatory protein regulated by SF-1, GATA-4 and C/EBP-beta in mouse Leydig tumor cells. Reprod Biol Endocrinol. (2013) 11:72. 10.1186/1477-7827-11-72 23889939 PMC3734203

[170] Fisher JS Macpherson S Marchetti N Sharpe RM. Human ‘testicular dysgenesis syndrome’: a possible model using *in-utero* exposure of the rat to dibutyl phthalate. Hum Reprod. (2003) 18:1383–94. 10.1093/humrep/deg273 12832361

[171] Xing JS Bai ZM Is testicular dysgenesis syndrome a genetic, endocrine, or environmental disease, or an unexplained reproductive disorder? Life Sci. (2018) 194:120–9. 10.1016/j.lfs.2017.11.039 29183799

[172] Rocca MS Di Nisio A Sabovic I Ghezzi M Foresta C Ferlin A. E2F1 copy number variations contribute to spermatogenic impairment and cryptorchidism by increasing susceptibility to heat stress. Andrology. (2019) 7:251–6. 10.1111/andr.12583 30659775

[173] Kristensen DG Nielsen JE Jorgensen A Skakkebaek NE Rajpert-De Meyts E Almstrup K. Evidence that active demethylation mechanisms maintain the genome of carcinoma *in situ* cells hypomethylated in the adult testis. Br J Cancer. (2014) 110:668–78. 10.1038/bjc.2013.727 24292451 PMC3915112

[174] Schagdarsurengin U Steger K. Epigenetics in male reproduction: effect of paternal diet on sperm quality and offspring health. Nat Rev Urol. (2016) 13:584–95. 10.1038/nrurol.2016.157 27578043

[175] Raghavan D. Testicular cancer: maintaining the high cure rate. Oncology (Williston Park). (2003) 17:218–28. Available online at: https://www.cancernetwork.com/testicular-cancer/testicular-cancer-maintaining-high-cure-rate 12632864

[176] Drögemöller BI Monzon JG Bhavsar AP Borrie AE Brooks B Wright GEB. Association between SLC16A5 genetic variation and cisplatin-induced ototoxic effects in adult patients with testicular cancer. JAMA Oncol. (2017) 3:1558–62. 10.1001/jamaoncol.2017.0502 28448657 PMC5824214

